# Supplementary material for: MetaRibo-Seq measures translation in microbiomes
Source: Nat Commun. 2020 Jun 29;11:3268. doi: 10.1038/s41467-020-17081-z (PMC7324362; doi:10.1038/s41467-020-17081-z)
Supplement: Supplementary file 10 — Supplementary Data 7 [file 41467_2020_17081_MOESM10_ESM.zip › File2/Confidence_VeryHigh_Taxonomy/86001_out.krona.html]

Javascript must be enabled to view this page.

members
magnitude
magnitudeUnassigned
count
unassigned
taxon
rank

86001\_out

32

1

SRS055017\_contig\_number\_contig-100\_19980.122203

superkingdom
2
30

SRS014683\_contig\_number\_19374SRS015890\_contig\_number\_contig-100\_1010.164180
2

28
1239
phylum

28
186801
class

order
186802
28
5

SRS015578\_contig\_number\_25059SRS015854\_contig\_number\_contig-100\_616.139805SRS016335\_contig\_number\_14452SRS019381\_contig\_number\_19878SRS098571\_contig\_number\_66683

31979
family
1

1485
genus
1

1

SRS019068\_contig\_number\_contig-100\_6371.543981
59620
species


SRS015431\_contig\_number\_contig-100\_2433.465117
1
22
186804
family

genus
1501226
2

1

SRS015890\_contig\_number\_contig-100\_924.164094
1776391
species

species
1507512

SRS142503\_contig\_number\_45994
1

19
genus
1849828


SRS014613\_contig\_number\_18879SRS015663\_contig\_number\_contig-100\_1649.202214SRS015960\_contig\_number\_contig-100\_566.566SRS017247\_contig\_number\_8683SRS018984\_contig\_number\_contig-80\_662.238530SRS020271\_contig\_number\_contig-100\_12317.12318SRS024549\_contig\_number\_1437SRS045713\_contig\_number\_contig-100\_3003.3003SRS046502\_contig\_number\_6446SRS053214\_contig\_number\_contig-100\_595.128471SRS054956\_contig\_number\_16881SRS056519\_contig\_number\_13677SRS065504\_contig\_number\_40120SRS075398\_contig\_number\_contig-100\_3057.3058SRS077730\_contig\_number\_18252SRS098881\_contig\_number\_contig-100\_464.179039SRS103987\_contig\_number\_34873SRS104036\_contig\_number\_6017SRS143070\_contig\_number\_contig-100\_4361.102925
19
species
1505

2759
superkingdom
1

kingdom
4751
1

451864
subkingdom
1

1
4890
phylum

147538
subphylum
1

1
147550
class

subclass
222543
1

1028384
order
1

1
681950
family

1
5455
genus

species
31870
1

SRS144297\_contig\_number\_contig-100\_20726.20727
